# Arterial Spin Labeled MRI to Detect Early Placental Perfusion Differences in Fetal Heart Disease

**DOI:** 10.1001/jamanetworkopen.2025.37282

**Published:** 2025-10-13

**Authors:** Rachel L. Leon, Marjorie Navalta, Joshua S. Greer, Ananth J. Madhuranthakam, Durga Udayakumar

**Affiliations:** 1 Department of Pediatrics, University of Texas Southwestern Medical Center, Dallas; 2 Philips Healthcare, Cincinnati, Ohio; 3 Department of Radiology, University of Texas Southwestern Medical Center, Dallas; 4 Department of Radiology, Mayo Clinic, Rochester, Minnesota

## Abstract

This cohort study evaluates the use of arterial spin labeling magnetic resonance imaging (ASL MRI) to quantify placental perfusion in pregnant individuals with a fetus with congenital heart disease.

## Introduction

Placental histopathologies frequently occur in pregnancies complicated by fetal congenital heart disease (CHD).^1^ Concomitant disturbances in placental hemodynamics may affect fetal development but typically remain undiagnosed until after pregnancy. Noninvasive, in vivo assessment of placental perfusion could offer real-time insight into placental function.^2^ Arterial spin labeling (ASL) magnetic resonance imaging (MRI), a noncontrast quantitative technique, enables perfusion measurement without exogenous contrast agents. Flow alternating inversion recovery (FAIR) ASL, previously validated in complex anatomical structures such as the lungs,^3^ may be well-suited for evaluating placental perfusion. In this study, we investigated the efficacy of FAIR-ASL MRI for quantifying placental perfusion in individuals with a fetus with CHD.

## Methods

This prospective pilot cohort study was approved by the University of Texas Southwestern Medical Center institutional review board and follows the STROBE reporting guideline, where applicable. We enrolled individuals in the US from October 2021 to September 2023 with a fetus with CHD or without CHD (healthy controls), excluding those with extracardiac anomalies or genetic conditions. Written informed consent was obtained. Imaging was performed in the third trimester on a 1.5 T MR scanner (Ingenia, Philips Healthcare) using an optimized FAIR sequence. We used linear mixed-effects models with random intercepts for repeats and fixed effects of CHD, gestational age (GA), maternal body mass index (BMI; calculated as weight in kilograms divided by height in meters squared), and placental position. Single scans at less than 32 weeks’ GA used linear regression. Missing data were imputed using predictive mean matching and pooled with Rubin rules. Significance was set as a 2-sided *P* < .05. Analyses were conducted using Prism version 10.3.1 (GraphPad) and R software version 4.4.3 (R Project for Statistical Computing). See the eMethods in Supplement 1 for more details.

## Results

**Figure 1.**
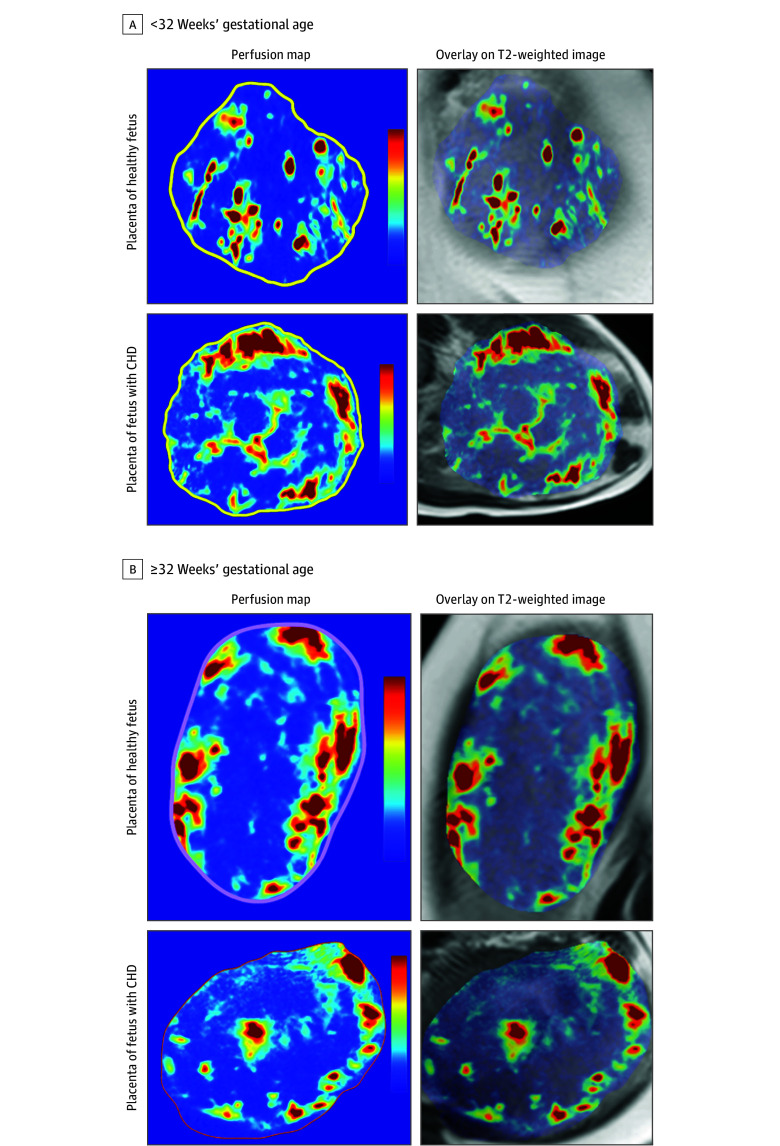
Representative Placental Perfusion Maps A, Representative quantitative perfusion maps (left) and perfusion maps overlaid on anatomical T2-weighted images (right) from an individual with a fetus without congenital heart disease (CHD) at 28 weeks 5 days’ gestation (top row) and an individual with a fetus with CHD (tetralogy of Fallot) at 29 weeks 2 days’ gestation (bottom row). B, Similar representative images from an individual with a fetus without CHD at 35 weeks 5 days’ gestation (top row) and an individual with a fetus with CHD (coarctation of the aorta with right-to-left ventricular size discrepancy, mild ventricular dilation, and hypertrophy) at 36 weeks’ gestation (bottom row). Color scale indicates perfusion values from 0 to 300 mL/min/100 g.

**Figure 2.**
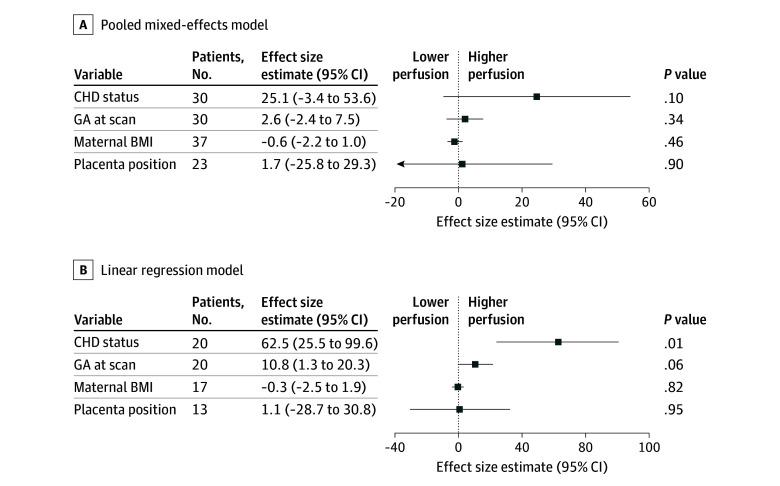
Estimated Associations of Congenital Heart Disease (CHD) and Clinical Covariates With Placental Perfusion Forest plots illustrating adjusted effect size estimates, which represent the mean difference in flow alternating inversion recovery arterial spin labeling perfusion signal for each variable, adjusted for all others in the model. Squares indicate point estimates; horizontal lines represent 95% CIs. Values to the right of the dashed line reflect relatively higher placental perfusion. Reference groups were healthy control for fetal CHD status and posterior for placental position; the continuous variables gestational age (GA; weeks) and body mass index (BMI; calculated as weight in kilograms divided by height in meters squared) were modeled per unit. A, Results from a pooled mixed-effects model in the whole cohort showing that there were no significant differences in placental perfusion observed between individuals with fetuses with CHD and individuals with fetuses without CHD or the other clinical variables tested. B, Results from linear regression model from magnetic resonance imaging scans obtained at less than 32 weeks’ GA showing higher placental perfusion in individuals with a fetus with CHD.

The study cohort comprised 20 pregnant individuals with healthy fetuses (median [range] maternal age, 31.5 [27.0-34.8] years, mean [SD] BMI, 33.3 [7.6]; 7 with male fetal sex [35%]; 10 with repeat imaging [50%]) and 10 people with a fetus with CHD (median [range] age, 24.0 [20.5-34.0] years; mean [SD] BMI, 28.3 [5.0]; 6 with male fetal sex, [60%]; 7 with left ventricular outflow tract obstruction [70%]; 2 with tetralogy of Fallot [20%]); 1 with double outlet right ventricle [10%]; 2 with repeat imaging [20%]) imaged at 24 to 38 weeks’ GA. Those with repeat imaging (12 individuals [40%]) had a minimum of 4 weeks between scans. Placental perfusion imaging was successful in all individuals (Figure 1). In the full cohort, fetal CHD was not significantly associated with placental perfusion (Figure 2A). However, in exploratory subgroup analysis of individuals imaged at less than 32 weeks’ GA, fetal CHD was associated with higher placental perfusion (estimate, 62.5 mL/min/100 g; 95% CI, 25.4 to 99.6 mL/min/100 g; *P* = .01) (Figure 2B).

## Discussion

Our findings demonstrate the capability of FAIR-ASL MRI to noninvasively quantify total placental perfusion. Elevated placental perfusion in individuals with a fetus with CHD before 32 weeks’ gestation requires validation in a larger cohort, but if confirmed, may represent a compensatory hemodynamic response to fetal hypoxia, a phenomenon previously reported.^4^ Because FAIR-ASL measures total placental perfusion, the elevations likely reflect augmented maternal blood flow to counteract reduced fetal oxygenation. Consequently, distinct CHD subtypes may exert differential effects on placental perfusion, depending on their impact on fetal oxygen delivery, and will be investigated in future studies. Negligible perfusion differences after 32 weeks’ GA align with previous findings in fetal CHD using alternative ASL methodologies^5^ and our absolute perfusion values are similar to those reported in larger cohorts.^6^ Early identification of altered placental perfusion in complicated pregnancies may facilitate timely interventions aimed at enhancing clinical outcomes. Despite inherent limitations of this preliminary investigation, including its modest sample size, our study underscores the potential of FAIR-ASL as a robust modality for placental perfusion assessment, and may be particularly informative in cases of fetal pathology.

## Notes

Supplement 1. eMethods. Imaging Specifications

Supplement 2. Data Sharing Statement
