# Supplementary material for: Arterial Spin Labeled MRI to Detect Early Placental Perfusion Differences in Fetal Heart Disease
Source: JAMA Netw Open. 2025 Oct 13;8(10):e2537282. doi: 10.1001/jamanetworkopen.2025.37282 (PMC12519309; doi:10.1001/jamanetworkopen.2025.37282)
Supplement: Supplement 1. — eMethods. Imaging Specifications [file jamanetwopen-e2537282-s001.pdf]

Supplemental Online Content

Leon RL, Navalta M, Greer J, Madhuranthakam AJ, Udayakumar D. Arterial spin labeled MRI to detect early placental perfusion differences in fetal heart disease. *JAMA Netw. Open*. 2025;8(10):e2537282. doi:10.1001/jamanetworkopen.2025.37282

eMethods.

This supplemental material has been provided by the authors to give readers additional information about their work.

© 2025 Leon RL et al. *JAMA Network Open*.

eMethods.

FAIR-MRI parameters

Pregnant individuals were imaged in supine position using respiratory bellows at 1 or 2 time points (separated by approximately 4 weeks). Imaging parameters included an inversion delay of 1.5 seconds, single-shot turbo spin echo readout, 4 background suppression pulses, 3 inflow saturation pulses, 3 to 5 slices, 10 control-label image pairs, repetition time of 3 seconds, echo time of 46 to 52 milliseconds, spatial resolution of 3 × 3 × 15 mm^3, and FOV field of view of 24 to 28 cm, in a coronal orientation. Proton-density weighted images were acquired for quantitative normalization.

Statistical Analysis

We analyzed a continuous placental perfusion outcome measured at one or two scans per participant. The primary objective was to estimate associations between perfusion and congenital heart disease (CHD) status, gestational age at scan (GA), maternal body mass index (BMI), and placental position while accounting for within-participant correlation. We summarized perfusion by group and GA and visualized perfusion versus GA with locally weighted smooths and 95% confidence bands, stratified by CHD status.

In our primary model, we fit linear mixed-effects models with a participant-specific random intercept to account for repeated scans. Fixed effects were CHD status (Control as the reference), GA (continuous, weeks), BMI (continuous, kg/m^2), and placental position (posterior vs anterior, with anterior as the reference). Models were estimated by restricted maximum likelihood. We report unstandardized coefficients, standard errors, 95% confidence intervals, and two-sided p-values.

© 2025 Leon RL et al. *JAMA Network Open*.

We also conducted exploratory analyses in a subgroup of patients imaged at <32 weeks' GA. Because many participants imaged before 32 weeks had only a single scan, we conducted a prespecified complementary analysis restricted to scans with GA <32 weeks using ordinary least-squares linear regression with the same fixed-effect structure as our primary model (CHD status, GA, BMI, placental position). Results are presented as coefficients with 95% confidence intervals and two-sided p-values. We addressed missing values using multiple imputation by chained equations (**mice**) under a missing-at-random assumption. For the primary mixed-effects analysis, we created five imputed datasets using predictive mean matching for continuous variables. The imputation model included the outcome (perfusion), all predictors (CHD status, GA, BMI, placental position), a scan indicator, and a participant identifier to preserve within-person structure. Each imputed dataset was analyzed with the mixed-effects model, and estimates and standard errors were combined using Rubin's rules. For the <32-week subgroup, we generated a separate set of five imputations and fit the linear models within each imputed dataset, pooling results as above.

© 2025 Leon RL et al. *JAMA Network Open*.
