# Supplementary material for: Arterial Spin Labeled MRI to Detect Early Placental Perfusion Differences in Fetal Heart Disease
Source: JAMA Netw Open. 2025 Oct 13;8(10):e2537282. doi: 10.1001/jamanetworkopen.2025.37282 (PMC12519309; doi:10.1001/jamanetworkopen.2025.37282)
Supplement: Supplement 2. — Data Sharing Statement [file jamanetwopen-e2537282-s002.pdf]

Data Sharing Statement

Leon. Arterial Spin Labeled MRI to Detect Early Placental Perfusion Differences in Fetal Heart Disease. *JAMA Netw Open*. Published October 13, 2025.
doi:10.1001/jamanetworkopen.2025.37282

Data
Data available: Yes
Data types: Deidentified participant data
How to access data: UT Southwestern Research Data Repository:
https://utsouthwestern.libguides.com/utswrdr
When available: With publication

Supporting Documents
Document types: None

Additional Information
Who can access the data: Any reasonable request
Types of analyses: Any purpose
Mechanisms of data availability: With signed data agreement
